# Classification and rates of adverse events in a Malawi male circumcision program: impact of quality improvement training

**DOI:** 10.1186/s12913-016-1305-x

**Published:** 2016-02-17

**Authors:** Pamela K. Kohler, Dorothy Namate, Scott Barnhart, Frank Chimbwandira, Beth A. Tippet-Barr, Tom Perdue, David A. Chilongozi, Lyson Tenthani, Oliver Phiri, Wezi Msungama, King K. Holmes, John N. Krieger

**Affiliations:** Department of Global Health, University of Washington, Seattle, USA; Department of Psychosocial & Community Health, University of Washington, Seattle, USA; International Training and Education Center for Health, Lilongwe, Malawi; US Centers for Disease Control and Prevention, Health Services Branch, Lilongwe, Malawi; Malawi Ministry of Health, Lilongwe, Malawi; Department of Medicine, University of Washington, Seattle, USA; Department of Urology, University of Washington, Seattle, USA

**Keywords:** Male circumcision, HIV prevention, Quality improvement, Adverse events, Malawi

## Abstract

**Background:**

Assessing safety outcomes is critical to inform optimal scale-up of voluntary medical male circumcision (VMMC) programs. Clinical trials demonstrated adverse event (AE) rates from 1.5 to 8 %, but we have limited data on AEs from VMMC programs.

**Methods:**

A group problem-solving, quality improvement (QI) project involving retrospective chart audits, case-conference AE classification, and provider training was conducted at a VMMC clinic in Malawi. For each identified potential AE, the timing, assessment, treatment, and resolution was recorded, then a clinical team classified each event for type and severity. During group discussions, VMMC providers were queried regarding lessons learned and challenges in providing care. After baseline evaluation, clinicians and managers initiated a QI plan to improve AE assessment and management. A repeat audit 6 months later used similar methods to assess the proportions and severity of AEs after the QI intervention.

**Results:**

Baseline audits of 3000 charts identified 418 possible AEs (13.9 %), including 152 (5.1 %) excluded after determination of provider misclassification. Of the 266 remaining AEs, the team concluded that 257 were procedure-related (8.6 AEs per 100 VMMC procedures), including 6 (0.2 %) classified as mild, 218 (7.3 %) moderate, and 33 (1.1 %) severe. Structural factors found to contribute to AE rates and misclassification included: provider management of post-operative inflammation was consistent with national guidelines for urethral discharge; available antibiotics were from the STI formulary; providers felt well-trained in surgical skills but insecure in post-operative assessment and care. After implementation of the QI plan, a repeat process evaluating 2540 cases identified 115 procedure-related AEs (4.5 AEs per 100 VMMC procedures), including 67 (2.6 %) classified as mild, 28 (1.1 %) moderate, and 20 (0.8 %) severe. Reports of AEs decreased by 48 % (from 8.6 to 4.5 per 100 VMMC procedures, *p* < 0.001). Reports of moderate-plus-severe (program-reportable) AEs decreased by 75 % (from 8.4 to 1.9 per 100 VMMC procedures, *p* < 0.001).

**Conclusions:**

AE rates from our VMMC program implementation site were within the range of clinical trial experiences. A group problem-solving QI intervention improved post-operative assessment, clinical management, and AE reporting. Our QI process significantly improved clinical outcomes and led to more accurate reporting of overall and program-reportable AEs.

## Background

Male circumcision reduces the risk of HIV acquisition by men through heterosexual intercourse by approximately 60 % [1–3], prompting public health programs to rapidly scale-up voluntary medical male circumcision (VMMC) services in countries with high HIV prevalence [4]. In sub-Saharan Africa, the current goal set forth by the World Health Organization (WHO) and UNAIDS is to circumcise 80 % of 15–49 year old males by delivering over 20 million circumcisions to avert more than 3 million new HIV infections by 2015; a further 8.4 million VMMC procedures are required to meet 2025 targets of 80 % coverage [5]. After adopting VMMC programming in 2011, Malawi was expected to complete over 2 million procedures by the end of 2015 [6], in a setting with limited health infrastructure; just 0.02 physicians and 0.28 nurse/midwives per 1000 individuals [7]. By the end of 2014, VMMC programs in Malawi had achieved only 8 % of the 2015 VMMC target [8]. There is a tremendous push to rapidly expand VMMC services in order to reach short-term and longer-term goals.

As VMMC delivery scales-up rapidly in resource-limited health systems, it is important to assess adverse events (AE) to inform safe and effective program implementation. Randomized clinical trials of VMMC demonstrated AE rates ranging from 1.5 to 8 % [1–3]. There are inconsistent data on AE rates in subsequent VMMC programs [9–11]. A review of VMMC task-shifting to studies revealed AE rates from <1 to 38 % [12], and Kenyan programs documented AE rates ranging from 1 to 18 % during early implementation [13, 14] to 3–7 % post scale-up [15]. No program safety data from Malawi VMMC services have yet been published [16].

Assuring high quality of delivered care represents a substantial challenge in implementation of proven HIV prevention interventions [16, 17]. Failure to make VMMC services safe and patient-centered will impede further scale-up of VMMC services. Monitoring to improve service quality is one of seven pillars in the Joint Strategic Action Framework to Accelerate the Scale-up of VMMC for HIV Prevention in Eastern and Southern Africa [18]. Quality improvement (QI) activities, particularly those that employ a structured, group problem-solving approach [19, 20], have improved quality of care in varied settings. We hypothesized that such approaches might improve HIV prevention services in resource-limited settings. The aim of this project was to employ a QI approach to assess quality and safety of VMMC services and improve safety outcomes at a VMMC program in Lilongwe, Malawi.

## Methods

### Study design

Utilizing a clinical case conference approach, a group problem-solving, QI project involving retrospective chart audits, case-conference AE classification, and provider training was conducted at a VMMC clinic in Lilongwe, Malawi. Evaluation of the QI project involved a quasi-experimental pre/post design.

### Population and setting

Bwaila VMMC Clinic is operated jointly by the University of Washington International Training and Education Center for Health (I-TECH) and the Malawi Ministry of Health (MOH). The US Centers for Disease Control and Prevention (CDC) in Malawi provides technical and programmatic support. The clinic opened in September 2012 and performs approximately 1000 VMMC procedures per month. The clinic is located at a district hospital near the city center in Malawi, where nurses and clinical officers are trained to provide VMMC services using the forceps-guided technique [21].

Of 894 clients undergoing VMMC in November 2012, 721 (81 %) returned for their scheduled 48-hour post-operative visits, 491 (55 %) returned for their 7-day visits, and 207 (23 %) returned for their 6-week visits. This was representative of client follow-up throughout the study period.

### Data collection, clinical case conferences, and AE classification

A multi-disciplinary team of seven clinicians, monitoring and evaluation staff, technical advisors, and program managers audited the first 3000 client records for initial review (the amount feasible in a 2-week audit). Charts consisted of standardized forms provided by the MOH. Information collected during the audit included client’s reported reason for seeking VMMC, clinician-surgeon name, duration of operation, and any indication of a possible AE. In charts where possible AEs were identified, the team further recorded any available clinical information including: detailed clinical notes, timing, severity, progression, treatment, and outcomes.

Each day during the 2-week period, a case conference was held with the entire VMMC program clinical team and the multi-disciplinary audit team. Consistent with previous clinical trial and the President’s Emergency Plan for AIDS Relief (PEPFAR) definitions, the team jointly classified each event as not related, possibly related, or definitely related to the VMMC procedure. Each possible or definitely related AE was further classified as: mild (requiring only reassurance, monitoring, or local wound care); moderate (requiring clinical support such as antibiotic medication or additional suturing); or severe (requiring return to the operating theater or hospitalization). Each case was also reviewed to assess whether any treatment provided was appropriate, and to determine lessons learned for future management. These methods are generally consistent with recommendations from the MOH and approaches used in two of the three randomized clinical trials of VMMC to prevent HIV infection [1, 2, 21].

During data collection and case conferences, providers were also queried regarding lessons learned, challenges in provision of care, and client flow was traced through the clinic. After baseline evaluation, clinicians and managers developed a QI plan to improve prevention, assessment, and management of AEs. A repeat audit was conducted 6 months later, and proportions and severity of AEs before and after the QI intervention were compared.

### Data analysis

Data were analyzed using STATA SE v11.0 (StataCorp, College Station, TX). Summary statistics were calculated, and comparisons of AEs before and after the intervention utilized chi-squared tests of proportions.

### Ethical considerations

Clients consented to VMMC services through written informed consent, and evaluation data were collected using routine program tools. The Malawi National Health Sciences Research Committee provided a letter of approval for program evaluation and dissemination, and the University of Washington Human Subjects Division determined that study activities did not meet the regulatory definition of research under 45 CFR 46.102(d).

## Results

### Baseline audit

During baseline audits, we reviewed 3000 charts. Although nurses and clinical officers are both trained to circumcise clients in Malawi, the clinical officers conducted >90 % of all surgeries. Client reason for presentation was only available for the first 2192 clients due to changes in MOH forms. Overall, 67 % reported seeking VMMC for HIV or sexually transmitted infection (STI) prevention. Hygiene (11 %), medical (5 %), personal (5 %), and religious (4 %) reasons were also cited. A small number indicated cancer prevention (Table 1).

**Table 1:** AE Chart Audit Results from a VMMC Program Lilongwe, Malawi (2012)

	First audit	Repeat audit	*p*-value
	*n* = 3000	*n* = 2540	
Reason for seeking MC (*n* = 2192)			
HIV/STI	1477 (67.4)	-	
Hygiene	246 (11.2)	-	
Medical	103 (4.7)	-	
Religious or Cultural	108 (4.9)	-	
Personal	104 (4.7)	-	
Other	154 (7.0)	-	
Possible Adverse Events	418 (13.9)	190 (7.5)	<0.001
Case Conference Determination:			
Related AE	257 (8.6)	115 (4.5)	<0.001

Of 3000 procedures, we initially documented 418 (13.9 %) possible AEs. As we began recording cases, we found that most AEs were recorded as “infections” (5.4 %), with varying descriptions ranging from, “mild inflammation,” to, “pus at the frenulum.” Nearly all of these, as well as some not identified as AEs or infections in the chart, were treated with antimicrobial drugs. The choice and dose of antibiotics was surprising (e.g., oral ciprofloxacin and metronidazole, plus intramuscular gentamicin), until we learned that the staff had attended training on STI management and we located the national guidelines for urethral discharge, which included all of these drugs. We also inspected drawers containing available antimicrobials and found that the only antimicrobials available at the clinic were those listed in the STI formulary.

### Pseudo-outbreak investigation

We used sequential client identification (ID) numbers to explore changes in rates over time. Spikes in rates of AEs were consistent across VMMC providers, however many cases appeared similar to the audit team. Although the spikes were not associated with a particular surgeon, they appeared to be related to the same clinician at the post-operative visit. While MOH forms did not have a space to enter post-operative visit clinician name or initials, the handwriting was easily recognizable to the audit team. We established a ‘case definition’ to explore these spikes as if it were an ‘outbreak investigation,’ where cases met all of the following conditions: same clinician handwriting, ‘infection’ diagnosed at post-operative day 7, treated with ciprofloxacin and metronidazole, and then ‘resolved’ at the next visit. We re-reviewed all ‘possible AE’ charts and found that 289 (69 %) of the 418 met this definition. Although the program employs more than 20 nurses and clinical officers, 152 (52.6 %) of these cases were from the same nurse and highly suspicious for misdiagnosed infection. Further evidence included two charts where a second nurse intervened and documented withholding antibiotics. During the audit process, clinicians would also ask the consulting surgeon to review patients identified as having possible infections. The surgeon found that none of these clients showed signs of infection, rather were displaying mild inflammation consistent with the healing process. We therefore excluded the 152 cases as misclassification of infection.

### AE rates and classification

Of the remaining 266 AEs, the team concluded that 257 (97 %) were procedure-related. Among the 257 procedure-related AEs (8.6 per 100 VMMC procedures), 6 (0.2 %) were classified as mild, 218 (7.3 %) were moderate, and 33 (1.1 %) were severe. Although some of the cases may have been mild in presentation, the severity classification system in use by VMMC programs assigns a severity level based on the intervention and not necessarily the presentation. Therefore even if the team assumed a mild presentation based on the clinical report, a patient treated with antimicrobials was, by definition, assigned a moderate AE.

### Clinical lessons learned

Case conference reviews concluded that 89 % of AEs were not treated appropriately; the majority of these were prescribed antibiotics for mild symptoms. Among 45 cases deemed not to be AEs during case review, 36 (80 %) had been prescribed antibiotics; 19 of 24 cases (79 %) designated as appropriately treated were given no antibiotics, and all 170/170 cases (100 %) inappropriately treated were given antibiotics (median two drugs per AE case).

### Structural factors contributing to AE rates and management

Several structural factors were found to contribute to the rates and misclassification of cases. Provider prescription practices for management of post-operative inflammation were consistent with national guidelines for urethral discharge, available antibiotics were the STI formulary, and providers reported feeling well-trained in surgical care but insecure in skills related to post-operative assessment and care. Wound care supplies, such as normal saline, irrigation kits, or gauze, were limited in availability or stored in another room, while antibiotics were in a nearby drawer.

### Data quality

Although not the primary assessment of this audit, a number of data-quality issues were identified. Client identification numbers were assigned as clients were entered into the national VMMC registers in the recovery room. The client ID number corresponded to the row number in the register, however in several cases, the clinician had incorrectly entered or reversed the digits of the ID number when moving to the next page (e.g., 2441 becomes 2401), resulting in a large number of clients with two different ID numbers, or different clients with the same ID number, and a program miscount of the number of procedures. Other common issues included: missing data as chart notes described signs and symptoms of AEs without checking the box indicating an AE was identified, database overwrite issues as duplicate ID numbers were entered, and version control with old versions of forms being printed as supply of current forms ran out.

### Quality improvement intervention

Clinical and program teams implemented a QI plan based on several identified challenges. The first strategy was grouped around post-operative assessment and management. Providers expressed a lack of understanding of what a normal healing adult circumcision should look like and a need for a refresher in basic wound care training. Also of concern was the limited availability of basic wound care supplies. Staff expressed a desire to prioritize patient reassurance and basic wound hygiene before giving antibiotics, and agreed to query a second clinician prior to prescribing antibiotics. Onsite technical in-service surgical training in circumcision technique, assessment of healing, and proper wound care was also provided. The second strategy involved improvements in data entry and classification of AEs. Providers agreed to continue the case conference process, reviewing each potential AE both for treatment and classification in order to enhance clinical practice.

### Subsequent audit to assess QI program

A repeat process 6 months after implementing the QI program evaluated 2540 charts and identified 190 clients (7.5 %) with possible AEs (Table 1). Subsequent discussion in case conferences classified 123 clients to have had an AE, including 115 considered procedure-related events (4.5 per 100 VMMC procedures) (Table 2). Of these 115 procedure-related AEs: 67 (2.6 %) were classified as mild, 28 (1.1 %) moderate, and 20 (0.9 %) severe. Program-reportable (moderate and severe) AEs totaled 48 (1.9 per 100 VMMC procedures). Severity distribution and overall number of reportable AEs were significantly reduced (both *p* < 0.001). The follow up audit also identified changes in specific AE types, with fewer cases of infection (5.4 % before vs. 1.8 % after) and a higher proportion of bleeding cases (0.3 % before vs. 1.5 %) after the intervention. Overall rates of total AEs had decreased by 48 % (from 8.6 to 4.5 per 100 VMMC procedures, *p* < 0.001) and moderate-plus-severe (program-reportable) AEs decreased by 75 % (from 8.4 to 1.9 per 100 VMMC procedures, *p* < 0.001).

**Table 2:** Characteristics of Related AEs Pre and Post-intervention in a VMMC Program Lilongwe, Malawi (2012)

	First audit	Repeat audit	*p*-value
	*n* = 3,000	*n* = 2,540	
Severity			
Mild	6 (0.2)	67 (2.6)	<0.001
Moderate	21 8 (7.3)	28 (1.1)	
Severe	33 (1.1)	20 (0.8)	
Reportable (Moderate & Severe)	251 (8.4)	48 (1.9)	<0.001
Type of AE			
Infection	161 (5.4)	46 (1.8)	<0.001
Hematoma	24 (0.8)	0 (0.0)	
Swelling	15 (0.5)	0 (0.0)	
Bleeding	10 (0.3)	37 (1.5)	
Wound disruption	2 (0.1)	16 (0.6)	
Delayed wound healing	12 (0.4)	3 (0.1)	
Damage to penis	3 (0.1)	1 (0.04)	
Insufficient skin removal	4 (0.1)	3 (0.1)	
Pain	0 (0.0)	5 (0.2)	
Urethral fistula	0 (0.0)	1 (0.04)	
Poor appearance	1 (0.03)	0 (0.0)	
Nonspecific or Unable to assess	23 (0.8)	0 (0.0)	
	First Audit n=257 (8.6 %)	Repeat Audit n=115 (4.5 %)	p-value
Appropriately treated	25 (9.7)	108 (93.9)	<0.001
Median (range) number of drugs/AE	2 (0–5)	1 (1–2)	<0.001

## Discussion

The initial review of the VMMC program caused concern because of a high rate of AEs compared to the rates observed in clinical trials (14 % compared to a range of 1–8 %). This concern led to a detailed review of 3000 initial patient charts on a line-by-line basis. We identified a ‘pseudo-outbreak’ that was not associated with particular VMMC surgical service providers, but with post-operative assessment and likely misclassification of normal events as “severe or moderate” AEs. Our detailed review identified a number of easily achievable changes that we targeted for our QI intervention. None of these activities targeted the surgical procedure, but rather the assessment, clinical management, and documentation of events.

The first area was to improve staff training and education on post-operative wound assessment and wound care. While wound care in general is a part of nursing and clinical officer curricula, adult male circumcisions are relatively new procedures to these cadres of clinicians. An emphasis on training for the surgical procedure is key and was generally well provided by our national program. However, we identified the need for additional staff training in assessment and management of post-operative wound care, with an emphasis on local wound care only for patients with mild postoperative inflammation or mild infections, such as a stitch abscess, rather than prescription of multiple antimicrobials.

The identified possible ‘wound infections’ routinely treated with multiple antimicrobials often were described as post-operative inflammation or other mild symptoms that would likely respond to local wound care alone. The Infectious Diseases Society of America’s updated 2014 guidelines for diagnosis and management of skin and soft tissue infections state that antimicrobials are not indicated for surgical site infections in the absence of fever or systemic symptoms [22]. Excessive use of antimicrobials is consistent with findings from other settings and with local clinical practice. While we aimed to decrease the number of antimicrobials prescribed, a few programs have reported isolated cases of Fournier’s gangrene after surgical circumcision [23], so there may be some benefit to more broad antimicrobial coverage in addition to secondary surgical management in highly selected cases. This must be balanced with concerns for promoting emergence of antibiotic resistance and over use of antibiotics.

The second area of improvement was related to AE documentation and classification. Most of the charts of moderate AE cases described no or mild symptoms, however the AE became classified as moderate due to the medical intervention of prescribing antimicrobials. A reporting classification system that determines AE severity based on the treatment provided, and not the symptoms assessed, will lead to increased rates of reported AEs if providers tend to rely on excessive use of antimicrobials. Classification systems that focus more specifically on assessment rather than treatment to categorize events will be useful for Malawi and other VMMC programs.

Finally, while there is no tradition of a “case conference for QI” in Malawi, the QI group-problem solving process appeared to provide a successful approach to improve quality of clinical care by engaging the clinical team in discussions of program outcomes. Our experience was that the team took ownership of program and that the use of data in program operations provided a framework for continued improvement.

This study has several strengths and limitations. This QI process was unique in that it included a detailed and thorough review of more than 5000 client charts, and utilized a team-based approach that bridged clinical and evaluation personnel for a common purpose. Several data quality issues were identified in the process of the audit, however we were not able to quantify these errors. While our repeat audit AE numbers are reassuring, a recent study from Kenya suggests that AE rates may be higher than reported given the context of low rates of client follow up in clinic (6.8 AE per 100 VMMC procedures in the lost to follow up group compared to 3.3 AE per 100 VMMC procedures in the adherent to follow up group) [15]. In our program, 7-day follow up rates are 55 %, suggesting the possibility of similar under-reporting in Malawi. Given the rapidly changing service delivery environment, attribution of the temporal improvement in AE rates to our QI process must be interpreted with caution. Furthermore, it is unclear how much of the improvement could be attributed to changes in post-operative clinical service versus improved data collection and AE classification.

## Conclusions

AE rates from this implementation site are within range of clinical trial experiences. However, we detected problems with post-operative assessment, clinical management, and documentation of AEs. Our QI process significantly decreased the rates of overall and of program-reportable AEs, resulting in improved clinical assessment and post-operative management, as well as more accurate program reporting. A similar process could benefit other VMMC programs. Lessons learned from this process are likely to apply to provision of VMMC services during VMMC rollout in other resource-limited settings.

## Abbreviations

AE: adverse event
CDC: centers for disease control and prevention
ID: identification
I-TECH: international training and education center for health
MOH: ministry of health
PEPFAR: President’s emergency plan for aids relief
VMMC: voluntary medical male circumcision
QI: quality improvement
STI: sexually transmitted infection
WHO: World Health Organization
